# Levosimendan for sepsis-induced myocardial dysfunction: friend or foe?

**DOI:** 10.3389/fcvm.2024.1520596

**Published:** 2025-01-08

**Authors:** Xinxin Du, Fang Xiong, Yafei Hou, Xiangyou Yu, Pengfei Pan

**Affiliations:** ^1^ Cardiac Intensive Care Center, Zhongshan Hospital, Fudan University, Shanghai, China; ^2^ Department of Critical Care Medicine, The First Affiliated Hospital of Xinjiang Medical University, Urumqi, Xinjiang, China; ^3^ Department of Critical Care Medicine, Chongqing University Three Gorges Hospital, Wanzhou, Chongqing, China; ^4^ Shanghai Institute of Immunology, Shanghai Jiao Tong University School of Medicine, Shanghai, China

**Keywords:** levosimendan, inotropes, sepsis, sepsis-induced myocardial cardiac dysfunction, organ protection

## Abstract

Sepsis-induced myocardial dysfunction (SIMD) involves reversible myocardial dysfunction. The use of inotropes can restore adequate cardiac output and tissue perfusion, but conventional inotropes, such as dobutamine and adrenaline, have limited efficacy in such situations. Levosimendan is a novel inotrope that acts in a catecholamine-independent manner. However, study results regarding the treatment of SIMD with levosimendan are inconsistent, and the use of levosimendan is highly controversial. In this review, we summarized the therapeutic mechanisms of levosimendan in SIMD and considered recent research on how to improve the efficacy of levosimendan in SIMD. We also analyzed the potential and limitations of levosimendan for the treatment of SIMD to provide ideas for future clinical trials and the clinical application of levosimendan in SIMD.

## 1 Introduction

Sepsis is characterized by life-threatening organ dysfunction caused by a dysregulated host response to infection (1). Despite the significant advances in the past few decades, sepsis and septic shock remain the leading causes of death in intensive care units (ICUs). In addition to the distributive shock caused by vascular hyporesponsiveness and autonomic nervous dysfunction, sepsis can induce myocardial depression and consequently reduce cardiac pumping and cardiac output (CO), which is manifested as treatment-resistant hypotension that responds poorly to fluid resuscitation and vasoactive agents. This acute reversible myocardial depression secondary to sepsis is known as sepsis-induced cardiac dysfunction (SIMD) or sepsis-induced cardiomyopathy (SICM). The use of inotropes can restore sufficient CO and peripheral blood oxygen delivery in SIMD patients (2). Digitalis, catecholamines, and phosphodiesterase (PDE) inhibitors increase myocardial contractility by increasing the levels of intracellular cyclic adenosine monophosphate and Ca^2+^. However, elevated intracellular Ca^2+^ levels increase myocardial oxygen consumption and predispose to arrhythmias.

Levosimendan is a distinctive inodilator that combines calcium sensitization, PDE inhibition, and vasodilatory properties through the opening of adenosine triphosphate (ATP)-dependent K^+^ channels (3). In 2000, it was first approved in Sweden for the short-term treatment of acutely decompensated severe chronic heart failure when conventional therapy is not effective and in cases where inotropic support is required (3). An increasing number of studies have shown that levosimendan improves cardiac function without affecting myocardial oxygen consumption and has protective effects on other organs. Therefore, levosimendan is an ideal treatment choice for sepsis complicated by myocardial depression. However, the use or non-use of levosimendan during sepsis and septic shock remains controversial. The current Surviving Sepsis Campaign (SSC) guidelines do not recommend levosimendan for the treatment of septic shock for several reasons: it is expensive, ineffective, not easily available, and can lead to hypotension and arrhythmias (4). Previous studies have shown conflicting results regarding the use of levosimendan in SIMD, and the timing and methods of its application also need to be investigated further. Whether levosimendan can be used in the treatment of SIMD is full of controversy, and many questions need to be answered regarding levosimendan in the treatment of SIMD.

## 2 SIMD: a significant global challenge

SIMD or SICM is a reversible myocardial dysfunction that occurs as part of multiple organ failure caused by sepsis and septic shock (5). There is no objective definition of SIMD. Although the definition of SIMD is based on left ventricular systolic dysfunction, both ventricles may be affected (6). Martin et al. (7) defined SIMD as an acute cardiac dysfunction syndrome associated with sepsis that is unrelated to myocardial ischemia, with one or more of the main clinical features: (1) left ventricular dilation with normal or reduced filling pressure; (2) reduced ventricular contractility; and (3) right ventricular diastolic dysfunction or left ventricular (systolic or diastolic) dysfunction with reduced volume responsiveness. For patients with sepsis-related organ dysfunction, particularly those with septic shock who require vasopressors, the possibility of SIMD should be considered (8). Based on the clinical and echocardiographic parameters, Geri et al. (9) classified septic shock into five phenotypes: (1) well resuscitated; (2) left ventricular systolic dysfunction; (3) hyperkinetic state; (4) right ventricular failure; and (5) hypovolemic. Patients with different echocardiographic manifestations may require different treatments and have different clinical outcomes. Zhang et al. (10) found that according to LVEF, patients with sepsis were divided into high LVEF group (LVEF higher than or equal to 70%), normal LVEF group (LVEF higher than or equal to 50% and less than 70%), and low LVEF group (LVEF less than 50%), and patients with low LVEF had the highest in-hospital mortality and 28-day mortality.

The epidemiology of SIMD remains elusive due to the lack of a consensus on its definition. According to previous studies, the prevalence of SIMD in sepsis and septic shock ranges from 18% to 40%, while in some reports it can reach as high as 70% (11). SIMD is increasingly recognized as a major and severe complication of sepsis and septic shock, posing a significant challenge globally. Patients with SIMD have a mortality rate of 70%–90%, which is 2–3-fold higher than that of non-cardiac affected septic patients (12). In a meta-analysis involving 1,373 patients with sepsis and septic shock, the incidence of right ventricular dysfunction was 35%, and the occurrence of right ventricular dysfunction was associated with higher short- and long-term mortality (13). With appropriate treatment, myocardial dysfunction may recover within 7–10 days (14).

Over the past 20 years, the mechanism of SIMD has been extensively studied in various fields, including proteomics and genomics (15). The pathophysiology of SIMD is complex, and its exact mechanisms need further investigation. The pathological mechanisms of SIMD include the release of bacterial endotoxins, mitochondrial dysfunction, and increased levels of cytokines, inflammatory mediators, nitric oxide (NO), and reactive oxygen species (16). These mechanisms decrease the intrinsic contractility of the heart in septic patients. In another review, Bi et al. (15) found that mechanisms such as cell apoptosis, mitochondrial damage, autophagy, excessive inflammatory response, oxidative stress, and pyroptosis are involved in sepsis-induced myocardial injury. Sepsis-induced myocardial cell death involves cell apoptosis, necrosis, mitochondrial-mediated necrosis, pyroptosis, iron necrosis, and autophagy (17). Recent studies have shown that non-coding RNAs (including microRNA, long-chain non-coding RNA, and cyclic RNA) play a crucial role in the development of myocardial dysfunction after sepsis (12).

Echocardiography is the preferred method for the diagnosis of myocardial dysfunction due to sepsis. SIMD is characterized by reduced left ventricular contractility eventually associated with left ventricular dilatation with or without right ventricle failure (11). Systolic function [i.e., left ventricular ejection fraction (LVEF)] is the first parameter affected in the diagnosis of SIMD. An LVEF of ≤50% and the presence of left ventricle dilatation are generally used as diagnostic criteria for SIMD (18). In addition to impaired left ventricular systolic function, left ventricular diastolic function and right ventricle are also involved in SIMD patients. Some echocardiographic parameters, such as LVEF, depend on the load conditions of the cardiovascular system, particularly the afterload, making it difficult to determine whether the observed cardiac dysfunction is due to myocardial dysfunction or due to hemodynamic disturbances occurring during septic shock (19). The global longitudinal strain measured by speckle echocardiography is more sensitive and specific than LVEF for the diagnosis of myocardial dysfunction. Cardiac biomarkers are also used to evaluate cardiac involvement in sepsis. Troponin I is a highly sensitive marker for myocardial injury, which can be elevated without evidence of myocardial ischemia (16). The other biomarkers include troponin T, brain natriuretic peptides (B-type natriuretic peptide and N-terminal pro-B-type natriuretic peptide), and others.

Despite extensive research on SIMD, there is a lack of guidelines for its treatment that can improve the prognosis of sepsis. The recommended treatment options include fluid resuscitation, vasoactive drugs (including dobutamine), β-blockers, levosimendan, and aortic balloon counter pulsation (16). Inotropes have the same hypothetical benefits as vasopressors for increasing CO, which can improve oxygen delivery to peripheral tissues (20). When low CO manifestations caused by myocardial dysfunction are identified, it is at the discretion of the physician to decide whether or not to use inotropes. In septic shock, inotropes should not be used as first-line therapy (21). Inotrope use is indicated only in patients with signs of tissue hypoperfusion due to a low CO induced by impaired cardiac function (22). Salvage, optimization, stabilization, and de-escalation have been introduced to describe the different phases of shock resuscitation, and inotropes should mainly be used in the optimization stage. As for the choice of inotropes, Backer et al. (22) recommended using a limited dose of dobutamine, followed by substituting or adding enoximone or milrinone, as appropriate, and then substituting or adding in cases of severe impairment. Heart rate control may be an option for certain patients (8). The key to successful treatment of septic shock is the early identification of hemodynamic changes. Bedside echocardiography can guide fluid resuscitation and hemodynamic optimization. After initial fluid resuscitation, the amount and speed of fluid replenishment should be dynamically adjusted based on the volume responsiveness. If hypotension persists, timely vasopressor therapy should be initiated with vasopressor and/or inotropic therapy adjusted according to the measures of CO and tissue perfusion (19). In a retrospective study conducted by Lan et al. (23), the use of echocardiography in patients with septic shock improved outcomes at 28 days. A study by Fu et al. (24) in 2022 also reported similar findings. Finally, SIMD patients who do not respond to medical management should be promptly provided with mechanical circulatory support, including veno-arterial extracorporeal membrane oxygenation (25).

## 3 Basic pharmacology and mechanism of levosimendan

**Figure 1:**
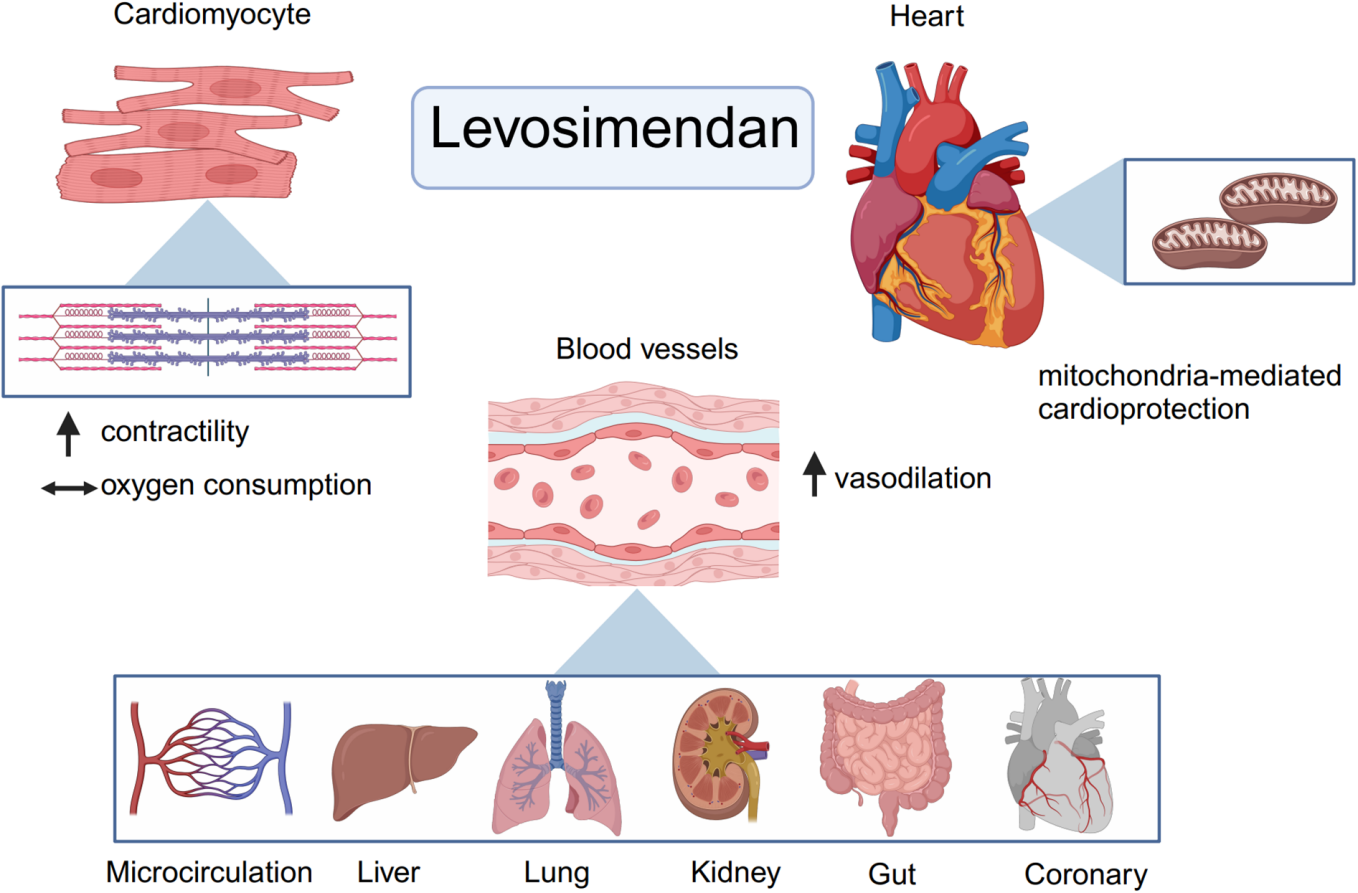
Basic pharmacology and mechanism of levosimendan. From a pharmacodynamic point of view, levosimendan has a threefold mechanism of action. First, as a calcium sensitizer, levosimendan can enhance myocardial contractility without increasing myocardial oxygen consumption. Then, levosimendan could open ATP- dependent K^+^ channels in the mitochondrial and cytoplasmic membranes of cardiomyocytes and thus has a cardioprotective effect. Finally, levosimendan could open ATP- dependent potassium channels in vascular smooth muscle cells to dilate blood vessels in other organs and improve organ perfusion such as kidneys, gut and so forth. (Created with BioRender.com).

Levosimendan is a novel calcium sensitizer and ATP-dependent K^+^ channel activator that is useful for the treatment of patients with acute decompensated heart failure and those requiring inotropic therapy. From a pharmacodynamic standpoint, levosimendan possesses a triple mechanism of action: (1) calcium sensitization by selective binding to Ca^2+^-saturated cardiac troponin C; (2) opening of ATP-dependent K^+^ channels in the cardiomyocyte mitochondria; and (3) opening of ATP-dependent K^+^ channels in vascular smooth muscle cells (3). The pharmacological mechanism of levosimendan is illustrated in Figure 1.

**Figure 2:**
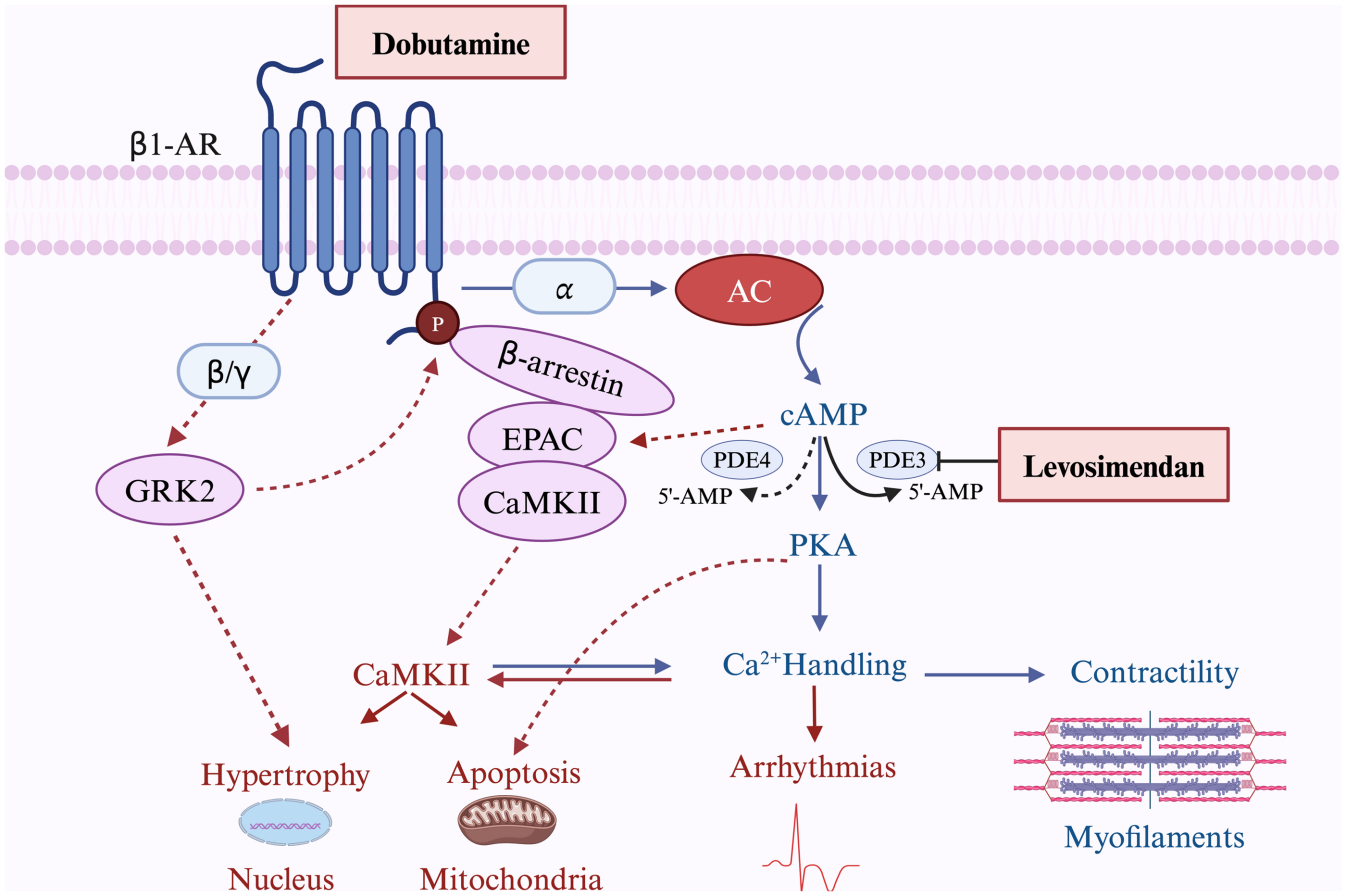
The pharmacological difference between levosimendan and dobutamine. The figure illustrates the distinct mechanisms of action of dobutamine and levosimendan. Dobutamine mainly activates β1-AR, leading to both beneficial effects (blue lines) through increased cAMP production, PKA activation, and enhanced calcium handling to improve contractility, and harmful effects (red lines) via CaMKII-mediated pathways promoting hypertrophy, apoptosis, and arrhythmias. Levosimendan enhances the cAMP-PKA pathway by inhibiting PDE3, amplifying beneficial effects. Solid lines represent direct actions, while dashed lines indicate indirect or secondary effects. β1-AR, Beta-1 Adrenergic Receptor; AC, Adenylyl Cyclase; cAMP, Cyclic Adenosine Monophosphate; PDE, Phosphodiesterase; PDE3, Phosphodiesterase 3; PDE4, Phosphodiesterase 4; PKA, Protein Kinase A; EPAC, Exchange Protein Activated by cAMP; CaMKII, Calcium/Calmodulin-Dependent Protein Kinase II; GRK2, G Protein-Coupled Receptor Kinase 2; 5’-AMP, 5’-Adenosine Monophosphate (Created with BioRender.com).

As a calcium sensitizer, levosimendan binds directly to troponin C, stabilizing the Ca^2+^-bound conformation of troponin, thus prolonging the actin-myosin interaction without altering cross-bridge cycling (26). This potentiating effect increases the interaction of actin and myosin at any given intracellular Ca^2+^ concentration, without significantly increasing the myocardial oxygen consumption. Levosimendan promotes the cardiac contractile force without an increase in the amplitude of intracellular Ca^2+^ transient (27). Furthermore, it exerts inotropic effects in a Ca^2+^-dependent manner. The gradual decline in intracellular Ca^2+^ concentration during diastole decreases the calcium-sensitizing effect of the drug, thereby preventing a postulated adverse influence on myocardial relaxation (28). In addition, levosimendan selectively inhibits PDE III, which has a positive lusitropic effect; this antagonizes the negative lusitropic effect of calcium sensitization (29). Overall, levosimendan enhances myocardial contractility without increasing myocardial oxygen consumption and improves diastolic function, which is theoretically superior to other inotropes such as dobutamine. Dobutamine and levosimendan exert their effects on the heart through distinct mechanisms. Dobutamine, a β1-AR agonist, increases cAMP production via adenylyl cyclase activation, leading to PKA-mediated enhancement of calcium handling and contractility. However, prolonged β1-AR activation also triggers harmful downstream effects, including hypertrophy, apoptosis, and arrhythmias, mediated by CaMKII and GRK2 pathways. The pharmacological differences between levosimendan and dobutamine are shown in Figure 2.

Levosimendan opens ATP-dependent K^+^ channels in the mitochondrial and cytoplasmic membranes of cardiomyocytes and thus has a cardioprotective effect in the presence of myocardial ischemia. The heart is highly dependent on high ATP levels to maintain its systolic and diastolic functions (30). Levosimendan improves mitochondrial Ca^2+^ overload, preserves high-energy phosphate, regulates the mitochondrial number, reduces infarct size, and mitigates myocardial ischemia/reperfusion injury (31, 32).

Levosimendan and its long-acting active metabolite OR-1896 activate multiple vasodilatory mechanisms, which involve the opening of ATP-sensitive K^+^ channels and other K^+^ channels as well as highly selective inhibition of the PDE III enzyme. Importantly, levosimendan does not inhibit PDE IV at low doses (33). Levosimendan increases myocardial oxygen supply by dilating coronary resistance vessels and improves tissue perfusion and oxygen metabolism in other organs by causing arterial and venous vasodilation. Notably, the plasma concentration at which levosimendan exerts its vasodilatory effects is far higher than that at which it exerts its inotropic effects.

In addition to its inotropic, cardioprotective, and vasodilatory effects, levosimendan may have protective effects on kidneys, liver, lungs, and gastrointestinal and central nervous systems through its anti-inflammatory, antioxidant, anti-apoptotic, and other effects (28).

The abovementioned pharmacological properties of levosimendan allow it to be widely used in critical illnesses in the ICU. In addition to acute decompensated heart failure, levosimendan is useful for chronic heart failure, perioperative prophylaxis of cardiac surgery, ventilator evacuation, and subarachnoid hemorrhage.

## 4 The rationale for levosimendan in the treatment of SIMD

Septic shock involves a complex interaction between abnormal vasodilation, relative or absolute hypovolemia, myocardial dysfunction, and altered blood flow distribution to the tissues (29). Early recognition and rapid reversal of tissue perfusion deficits due to infection are critical in the treatment of septic shock. Fluid administration, vasopressors, and inotropes aim to restore impaired tissue perfusion during septic shock. SIMD is mediated by multiple factors. However, regardless of the mechanisms, myocardial dysfunction is the main reason for the use of inotropes during septic shock. Dobutamine and other inotropes have typically been used to increase CO and oxygen transport, aiming to restore cell respiration and aerobic metabolism (29). According to the 2016 SSC guidelines, the use of inotropes, such as dobutamine (up to 20 μg/kg·min^−1^), should be considered in patients with myocardial dysfunction (manifested as low CO, increased filling pressures, and persistent tissue hypoperfusion) after adequate fluid resuscitation and use of vasopressors (34). The current SSC guidelines recommend the use of norepinephrine and dobutamine or adrenaline alone for patients with sufficient volume and blood pressure but persistently insufficient tissue perfusion (4). However, the use of dobutamine in SIMD remains controversial. Although dobutamine leads to an increase in cardiac index, myocardial oxygen demand also increases, thus increasing the risk of myocardial ischemia and tachyarrhythmias (35). The new inotrope levosimendan is a calcium sensitizer, which can improve myocardial function without affecting calcium ion flow. Unlike other inotropic agents, the positive inotropic effect of levosimendan is independent of ATP production; therefore, it can minimize oxygen demand, arrhythmias, and catecholamine resistance (36). Although the current SSC guidelines do not recommend the use of levosimendan, it has shown potential advantages in the treatment of SIMD.

Levosimendan can increase myocardial contractility. The CO level is normal or even high in septic patients after initial fluid resuscitation, but myocardial contractility may be impaired in a significant proportion of septic patients. During sepsis or under lipopolysaccharide (LPS) exposure, the Ca^2+^ homeostasis of myocardial cells is usually altered, leading to a decrease in myocardial contractility. Reduced systolic force limits the ability of the ventricle to reach a low end-systolic volume, resulting in a decrease in stroke volume. Nevertheless, the decrease in the stroke volume may be compensated by the increased end-diastolic volume achieved through adequate fluid resuscitation and by the decreased afterload due to arterial vasodilation (29). As a new calcium sensitizer, levosimendan increases the sensitivity of cardiomyocytes by altering the structure of troponin C, which can enhance myocardial contractility without increasing the intracellular calcium load or intracellular ATP level (18). Previous studies have shown that levosimendan increases myocardial contractility and CO, and improves tissue perfusion in septic patients (37). The use of other positive inotropic drugs is associated with side effects, including increased myocardial oxygen consumption, while levosimendan acts in a catecholamine-independent manner without increasing the myocardial oxygen consumption and heart rate.

Levosimendan can improve ventricular diastolic function. Diastolic dysfunction also occurs during sepsis and septic shock, which affects ventricular filling (38). Unlike the ATP-dependent inotropes milrinone and dobutamine, which can only improve the systolic function, levosimendan does not cause ventricular diastolic dysfunction due to Ca^2+^ overload and can improve ventricular diastolic function by improving the diastolic velocity ratio, shortening the diastolic phase, and improving the diastolic filling. In the experimental model of sepsis, Barraud et al. (39) found that levosimendan was superior to dobutamine and milrinone in restoring the ventricular systolic and diastolic functions of LPS-treated rabbits.

Levosimendan has several beneficial effects on hemodynamic parameters. Cardiovascular failure due to sepsis also involves peripheral vascular dysfunction. Abnormalities in the distribution of vital blood flow to the tissues may persist after the optimization of the CO. Levosimendan is a new positive inotropic drug with vasodilatory effects. Most previous studies have shown that levosimendan has beneficial effects on the macro-hemodynamics and tissue perfusion indices. Compared with dobutamine, levosimendan provides a favorable hemodynamic response without increasing the cardiac oxygen demand (40). Meng et al. (41) explored the effects of levosimendan on myocardial injury and systemic hemodynamic biomarkers in patients with septic shock. Compared with dobutamine, levosimendan reduced the biomarkers of myocardial injury, improved systemic hemodynamics in patients with septic shock, and reduced the duration of mechanical ventilation and ICU stay. The beneficial effect of levosimendan on microvascular distribution is independent of its effect on CO. Levosimendan can improve the sublingual microcirculation blood flow in patients with septic shock (42).

Apart from its direct effect on cardiac function, levosimendan also demonstrates multifaceted effects targeting specific pathological physiological mechanisms associated with SIMD. Levosimendan has anti-inflammatory and anti-apoptosis effects, improves myocardial ischemia, increases the synthesis of NO, protects vascular endothelial cells, and inhibits the expression of the hypoxia-inducible factor-1a (18). Moreover, levosimendan reduces the inflammatory response by downregulating nuclear factor-*κ*B-dependent transcription, inhibiting inducible NO promoter activity, and reducing NO expression *in vitro* (43). Sepsis can cause structural damage to myocardial mitochondria and loss of mitochondrial function. In a mouse model of myocardial injury caused by an intraperitoneal injection of LPS, Shi et al. (44) found that levosimendan inhibited inflammation and oxidative stress, and protected against LPS-induced cardiac dysfunction through mitophagy and the PINK-1-Parkin signaling pathway.

## 5 Application and limitations of levosimendan in septic shock and SIMD

After more than 20 years, the clinical applications of levosimendan in the field of emergency and critical care medicine have expanded considerably and now include various cardiac diseases (such as cardiogenic shock, Takotsubo cardiomyopathy, advanced heart failure, right ventricular failure, pulmonary hypertension, and cardiac surgery) and non-cardiac diseases (such as amyotrophic lateral sclerosis) (32).

In the clinical research on the use of levosimendan for septic shock and SIMD, early randomized controlled trials (RCTs) had small sample sizes. In 2015, a meta-analysis was conducted by Zandrillo et al. (45), which included 7 RCTs and 246 patients with severe sepsis or septic shock. Compared with conventional inotropes (mainly dobutamine), levosimendan significantly reduced the mortality, increased the cardiac index, and reduced the blood lactate level, but there was no difference in the mean arterial pressure or norepinephrine dose between the two groups. However, the data on the use of levosimendan in definite SIMD remains limited. Gordon et al. (46) published the results of a multicenter, randomized, double-blind, placebo-controlled clinical trial (LeoPARDS study) in The New England Journal of Medicine in 2016. The study had the largest sample size to date of any studies conducted on this topic. In this study, 516 patients with septic shock were randomly divided into the levosimendan and placebo groups. The results showed that levosimendan did not reduce the Sequential Organ Failure Assessment score and the 28-day mortality rate during hospitalization, but was associated with high risks of supraventricular tachyarrhythmia and ventilator weaning failure. Following that, Antcliffe et al. (47) conducted a subgroup analysis of the LeoPARDS study according to the levels of cardiac troponin I and NT pro-brain natriuretic peptide and failed to observe any benefit of levosimendan or decrease in the levels of inflammatory biomarkers in any subgroup. However, these research findings do not definitively refute the effectiveness of levosimendan in SIMD. As described in the LeoPARDS study, the cases selected did not undergo cardiac ultrasound and the proportion of patients with cardiac dysfunction was low, which may explain the lack of positive results (48). Putzu et al. (49) believe that the subjects included in the LeoPARDS study were low-risk patients in the relatively late stages of septic shock, who were not confirmed to have concomitant cardiac dysfunction, and who developed hypotension and supraventricular tachycardia after receiving high doses of levosimendan, up to 0.2 μg/(kg·min). Cardiac ultrasound examination or hemodynamic monitoring was not performed during the study, and no effective fluid resuscitation was performed before the administration of levosimendan. Overall, the LeoPARDS study tells us that septic patients with reduced systemic vascular resistance but no clinical signs of myocardial dysfunction do not benefit from levosimendan.

In a meta-analysis of 10 studies and 1,036 patients with sepsis and septic shock, levosimendan was more effective in reducing lactate levels (37). Compared with dobutamine, patients who received levosimendan reported significantly higher cardiac index (37). In another meta-analysis, levosimendan could reduce serum lactate levels more effectively and improve cardiac function (50). However, statistical significance in mortality was obscured after the LeoPARDS study was included. Interestingly, patients who received levosimendan also received significantly more fluid than their counterparts probably due to the vasodilatory effect of levosimendan. To sum up, these benefits have not been translated to the clinical endpoints in the two studies above.

Based on the results of the LeoPARDS study, the latest SSC guideline panel issued a weak recommendation against the use of levosimendan because of its lack of clinical benefits (4). The other reasons for not recommending its use include its safety profile, cost, and limited availability (4). However, these reasons should not negate the clinical application of this drug. The first concern with the use of levosimendan is its clinical benefit. The current SSC guidelines were directly influenced by the LeoPARDS study. As mentioned above, there are many concerns regarding the results of the LeoPARDS study, and the presence of clear evidence of myocardial dysfunction is required for levosimendan use. The second concern is drug safety. Levosimendan has a predictable safety profile, with the most common adverse events being hypotension, headache, and atrial arrhythmias (3). The 2021 European Society of Cardiology guidelines on chronic and acute heart failure suggest that in patients treated with β-blockers, levosimendan or a phosphodiesterase type 3 inhibitor may be superior to dobutamine because they act through independent mechanisms (51). Excessive peripheral vasodilatation and hypotension may be major limitations of levosimendan, especially when high doses are administered and/or bolus doses are initiated. However, at therapeutic doses, levosimendan is less likely to cause ventricular arrhythmias and can improve myocardial injury caused by sepsis (18). Hypotension associated with vasodilation can be prevented by adequate fluid resuscitation before levosimendan use, avoidance of loading doses, and concomitant use of vasopressors (2). The third issue is the cost of levosimendan. Levosimendan has a prolonged duration of action, with the drug effect lasting for over 7 days when the drug is administered intravenously for 24 h, whereas the other inotropes have a short duration of action. A prolonged duration (5–7 days) of intravenous administration of dobutamine is not associated with a lesser cost than 24 h of intravenous administration of levosimendan. The final issue is drug accessibility. The limited availability of the drug is not a valid reason for not recommending its use. With economic development, levosimendan is increasingly available even in low-income countries and regions.

A meta-analysis of 6 RCTs and 192 patients showed that, compared with dobutamine, the administration of levosimendan for 24 h significantly improved the cardiac index and left ventricular beat index, as well as significantly decreased the blood lactate level, in patients with SIMD, although there was no effect on the mortality rate or LVEF (31). In our previous meta-analysis, which included 10 RCTs and 543 patients with SIMD (defined as LVEF ≤ 50%), levosimendan increased the LVEF, decreased the cardiac troponin I and blood lactate levels, and reduced the mortality rate compared to dobutamine (52). In a recent prospective, single-blind, RCT, Sun et al. (53) enrolled 30 patients with severe SIMD (LVEF ≤ 35%) and compared the hemodynamic and clinical outcomes of patients treated with fixed doses of levosimendan (0.2 μg/kg·min^−1^) or dobutamine (5 μg/kg·min^−1^) for 24 h. The results showed that at 24 h, the cardiac index, LVEF, stroke volume index, and fluid volume were higher, whereas the norepinephrine dose was lower, in the levosimendan group than in the dobutamine group. On the third day, the cardiac troponin I level was lower in the levosimendan group than in the dobutamine group. There were no significant differences in terms of the 28-day mortality rate, length of ICU stay, and cost of ICU stay between the two groups, although the ventilator time was significantly shorter in the levosimendan group. A meta-analysis published in 2023 indicates that levosimendan is safe and effective in treating sepsis and septic cardiomyopathy (54). Recent evidence suggests that levosimondan significantly improves CI and lactate levels in patients with sepsis (55). However, due to the small sample sizes, inconsistent diagnostic criteria as well as high heterogeneity in the clinical studies included in the meta-analysis, conclusions drawn from it should be approached with caution. Most importantly, well-designed clinical trials are needed to determine the value of levosimendan in septic shock and SIMD. Main randomized controlled trials on the use of levosimendan in SIMD are reported in Table 1. It is noteworthy that no adverse events associated with levosimendan were reported in these RCTs. Upon detailed examination of the original studies, the absence of adverse events appears to reflect the actual trial outcomes rather than an omission or lack of data collection. The lack of adverse events in these RCTs may be attributed to factors such as stringent patient selection criteria or well-controlled dosing regimens. However, it is important to interpret this finding with caution. The relatively small sample sizes and the specific characteristics of the study populations may limit the generalizability of these results.

**Table 1:** Main randomized controlled trials on the use of levosimendan in sepsis-induced myocardial dysfunction.

Study	Year	Participants	Sample size (L/C)	Levosimendan therapy	Control therapy	Hemodynamic improvement	Mortality outcome	Potential adverse events	Conclusion
Morelli (56)	2005	Septic shock with LVEF ≤ 45% and PAOP ≥ 12 mmHg	15/13	Levosimendan (0.2 μg/kg·min^−1^) without a bolus loading dose for 24 h	Dobutamine (5 μg/kg·min^−1^) for 24 h	Improved	No reduction	Not reported	Favors levosimendan
Vaitsis (57)	2009	Severe sepsis or septic shock with CI < 2.2 or LVEF < 35%	23/19	Levosimendan (0.1 μg/kg·min^−1^) without a bolus loading dose for 24 h	Dobutamine (5–10 μg/kg·min^−1^) for 24 h	Improved	Reduced	Not reported	Favors levosimendan
Fang (58)	2014	Septic shock with LVEF ≤ 45%	18/18	Dobutamine (5 μg/kg·min^−1^) for 24 h; levosimendan (0.2 μg/kg·min^−1^) without a bolus loading dose for 24 h subsequently	Dobutamine (5 μg/kg·min^−1^) for 24 h	Improved	No reduction	Not reported	Favors levosimendan
Meng (41)	2016	Septic shock with LVEF ≤ 45%	19/19	Levosimendan (0.2 μg/kg·min^−1^) without a bolus loading dose for 24 h	Dobutamine (5 μg/kg·min^−1^) for 24 h	Improved	No reduction	Not reported	Favors levosimendan
Xu (59)	2018	Septic shock with LVEF ≤ 50%	15/15	Levosimendan (0.2 μg/kg·min^−1^) without a bolus loading dose for 24 h	Dobutamine (5 μg/kg·min^−1^) for 24 h	Improved	No reduction	Not reported	Favors levosimendan
Sun (53)	2023	severe SIMD (LVEF ≤ 35%)	15/15	Levosimendan (0.2 μg/kg·min^−1^) without a bolus loading dose for 24 h	Dobutamine (5 μg/kg·min^−1^) for 24 h	Improved	No reduction	Not reported	Favors levosimendan
Sun (60)	2024	Sepsis with LVEF ≤ 45%	20/20	Levosimendan (0.2 μg/kg·min^−1^) without a bolus loading dose for 24 h	Dobutamine (5 μg/kg·min^−1^) for 24 h	Improved	Not reported	Not reported	Favors levosimendan

CI, cardiac index; L/C, levosimendan group/control group; LVEF, left ventricular ejection fraction; PAOP, pulmonary arterial occlusion pressure; SIMD, sepsis-induced myocardial dysfunction.

## 6 Conclusions and perspectives

As one of the most serious complications of sepsis, SIMD has received extensive attention in recent years. Despite the SSC guideline recommends against the use of levosimendan for sepsis patients with cardiac dysfunction under certain conditions, it is crucial to recognize that recommendations should not override a clinician's individualized decision-making for specific cases. What's more, the guidelines do not explicitly oppose the administration of levosimendan in septic shock patients with confirmed low cardiac output syndrome (LCOS) by echocardiography, pulmonary artery catheter, or pulse index continuous cardiac output (61). In addition, as Vincent points out, a balance should be maintained between SSC guidelines and individualized care (62). With the development of precision medicine, identifying SIMD patients with certain treatable characteristics and giving targeted treatment may help to find patients who can benefit from levosimendan.

In conclusion, the present comprehensive review has highlighted key findings and insights into levosimendan for SIMD. The accumulated evidence underscores the significance of indications and timing of levosimendan administration. Moving forward, several avenues for future research merit attention. Exploring the pathogenesis of SIMD, establishing the gold standard for the diagnosis, and identifying subgroups of patients with different clinical manifestations of SIMD will contribute to a deeper understanding of SIMD. Furthermore, it is essential to integrate treatment and monitoring, and to equilibrate a delicate balance between clinical guidelines and the unique needs of individual patients. Embracing these future directions promises to advance the field and provide valuable implications for the application of levosimendan in SIMD.
